# Spatial response resampling (SR^2^): Accounting for the spatial point spread function in hyperspectral image resampling

**DOI:** 10.1016/j.mex.2023.101998

**Published:** 2023-01-02

**Authors:** Deep Inamdar, Margaret Kalacska, Patrick Osei Darko, J. Pablo Arroyo-Mora, George Leblanc

**Affiliations:** a Applied Remote Sensing Laboratory, Department of Geography, McGill University, Montréal, QC H3A 0B9, Canada; b Flight Research Laboratory, National Research Council of Canada, Ottawa, ON K1A 0R6, Canada

**Keywords:** Spatial resampling, Simulation, Data cross-validation, Flight planning, Data fusion, Point spread function, Spatial response, Pushbroom, MATLAB, Spatial Response Resampling (SR^2^)

## Abstract

With the increased availability of hyperspectral imaging (HSI) data at various scales (0.03–30 m), the role of simulation is becoming increasingly important in data analysis and applications. There are few commercially available tools to spatially degrade imagery based on the spatial response of a coarser resolution sensor. Instead, HSI data are typically spatially degraded using nearest neighbor, pixel aggregate or cubic convolution approaches. Without accounting for the spatial response of the simulated sensor, these approaches yield unrealistically sharp images. This article describes the spatial response resampling (SR^2^) workflow, a novel approach to degrade georeferenced raster HSI data based on the spatial response of a coarser resolution sensor. The workflow is open source and widely available for personal, academic or commercial use with no restrictions. The importance of the SR^2^ workflow is shown with three practical applications (data cross-validation, flight planning and data fusion of separate VNIR and SWIR images).

• The SR^2^ workflow derives the point spread function of a specified HSI sensor based on nominal data acquisition parameters (e.g., integration time, altitude, speed), convolving it with a finer resolution HSI dataset for data simulation.
• To make the workflow approachable for end users, we provide a MATLAB function that implements the SR^2^ methodology.

## Graphical abstract

**Figure:**
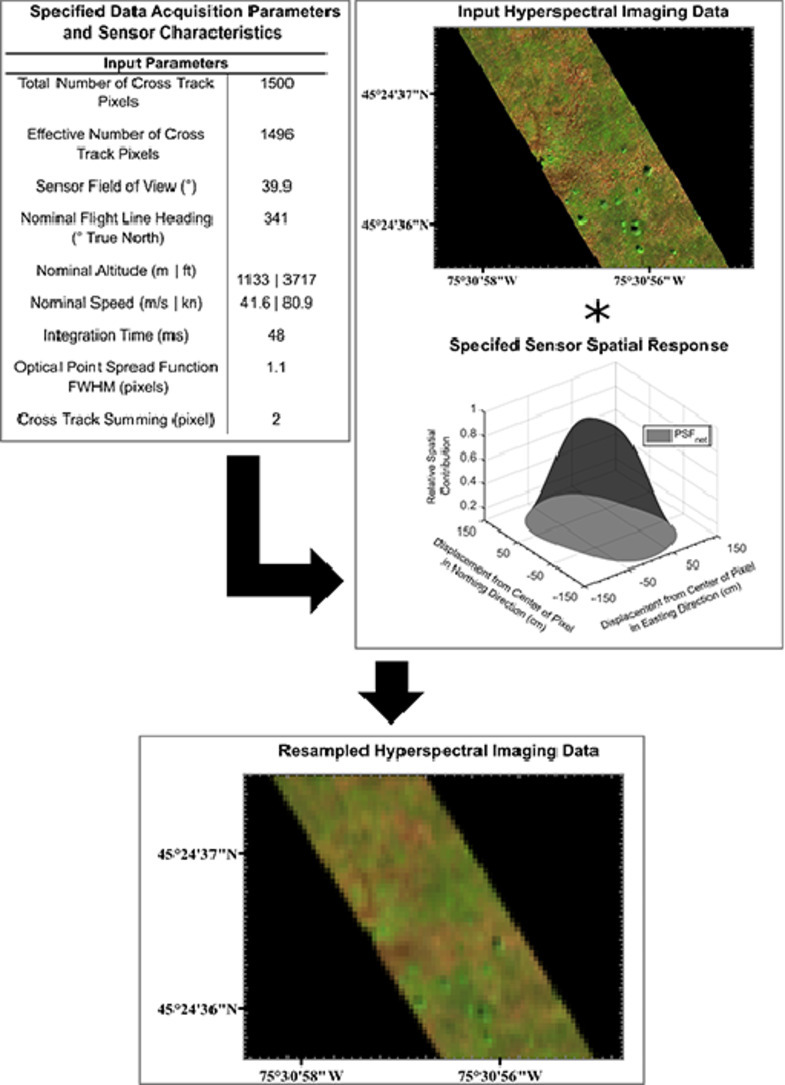

Specifications table

**Table:** 

Subject Area:	Earth and Planetary Sciences
More specific subject area:	Remote Sensing Data Processing
Method name:	Spatial Response Resampling (SR^2^)
Name and reference of original method:	**Data Simulation Approach:** Inamdar, D., Kalacska, M., Leblanc, G., & Arroyo-Mora, J.P. (2020). Characterizing and mitigating sensor generated spatial correlations in airborne hyperspectral imaging data. *Remote Sensing, 12*, 641 **MATLAB Function:** Inamdar, D., Kalacska, M., Leblanc, G., & Arroyo-Mora, J.P. (2021). Implementation of the directly-georeferenced hyperspectral point cloud. MethodsX, 8, 101429
Resource availability:	MATLAB 2020B with Hyperspectral Imaging Library from Image Processing Toolbox

## Method details

### Background

Over the past three decades, the abundance of spatial-spectral information captured by remotely sensed hyperspectral imaging (HSI) data has been actively exploited for various applications [1]. The utility of HSI data will only increase as remotely piloted aerial system (RPAS) (e.g., HySpex Mjolnir [2], ITRES μCASI-1920 [3], Specim Aisa KESTREL 10 [4], etc.), airborne (e.g., AVIRS-NG [5], ITRES CASI [6], APEX [7]) and spaceborne (e.g., DESIS [8], SHALOM [9], Carbon Mapper [10], EnMAP [11], PRISMA [46]) imagers become more prevalent. With the increased availability of optical remotely sensed data at various spatial and spectral resolutions, the role of simulation is becoming increasingly important in data analysis and application. Specifically, data simulation is a valuable tool for sensor optimization and development [12], flight planning [13], data cross validation and calibration [14] and algorithm development [15], amongst others. Many of the popular remote sensing data analytics software (e.g., ENVI (Harris Geospatial Solutions inc., Broomfield, CO, USA), CATALYST Professional (PCI Geomatics, Markham, Ontario, Canada) provide resampling tools to simulate the spectral characteristics of a coarser spectral resolution sensor based on its spectral response. In the spatial domain, HSI data simulation is carried out through spatial resampling algorithms. In addition to classical approaches (e.g., pixel aggregate, nearest neighbor, bilinear and cubic convolution), there are a variety of cutting-edge resampling techniques, especially in the data fusion literature, that have been effectively applied to HSI data (refer to the review papers by Mookambiga and Gomathi [16] and Yokoya et al. [17] for examples). In general, novel resampling techniques in the literature have concentrated on enhancing the spatial resolution of HSI data with finer resolution multispectral and panchromatic data sources. For instance, Lu et al. [18] developed a data fusion method based on convolutional neural networks to resample coarse resolution HSI data to the spatial resolution of a finer resolution panchromatic image. When spatially degrading HSI data via spatial resampling, classical techniques are more commonly used in the remote sensing literature.

Although the described spatial resampling approaches can simulate imagery at any desired pixel size, it is important to recognize that pixel size does not accurately represent the spatial characteristics of the spectrum collected by coarser resolution imagers. The spatial response of a sensor can be described by the spatial point spread function (PSF). The PSF maps the relative response of a single pixel as a function of displacement from the center of the pixel [19]. Hyperspectral PSFs generally follow a Gaussian shape. Correspondingly, the spectrum from any given pixel is not equally representative of the materials within its conventionally square pixel boundaries. A substantial portion of the signal to each pixel (> 40%) originates from materials outside the pixel boundaries defined by the spatial resolution [19]. When spatially resampling imagery for data simulation, it is not appropriate to use a simple average or a nearest neighbor approach as the characteristics of the simulated sensor are ignored. Instead, spatial resampling should be conducted using a weighted average based on the PSF of the simulated sensor. For instance, in the end-to-end simulation approach described by Blonski et al. [20], the spatial response of the simulated sensor was accounted for by convolving a synthetic HSI scene with the spatial PSF. Inamdar et al. [19] adopted a similar approach, convolving a theoretically derived PSF with a synthetic dataset to understand the importance of the spatial response. In this work, the overlap in the PSF of adjacent pixels was shown to lead to image blurring that reduced the natural spatial and spectral variance of the simulated scene. It is important to capture this loss of variance in simulation efforts. Otherwise, the simulated imagery will be unrealistically sharp, affecting downstream applications. For example, without accounting for the sensor PSF in flight planning efforts, simulated imagery might detect features of interest that cannot be observed in real imagery. This may lead end users to select inappropriate data acquisition parameters during aerial campaigns. Without accounting for sensor PSFs, it can also be difficult to compare, combine and apply imagery collected across various spatial scales. This is problematic given the increased availability of RPAS, airborne and spaceborne HSI data [21,22]. For instance, in many applications, it is desirable to have full-range HSI data. Due to technological and monetary restrictions, collecting full-range HSI data from a single sensor at a high spectral resolution is not generally feasible. Instead, the spectra from separate HSI datasets covering different portions of the electromagnetic spectrum need to be fused into one coherent signal. To ensure that the spectra from various data are optimally fused, it is critical to account for discrepancies in spatial scale between the utilized sensors. In this process, one image must be spatially degraded to match the spatial characteristics of the other image. Without accounting for discrepancies in spatial properties, any derived full-range spectrum would be unusable in conventional spectroscopy analyses such as material identification and characterization.

Expanding on the methodology implemented by Inamdar et al. [19], the objective of this study was to develop a spatial resampling workflow that accounts for the spatial response of a specified sensor. The developed spatial response resampling (SR^2^) workflow degrades georeferenced raster HSI data to the spatial characteristics of a coarser resolution sensor. In this workflow, the net PSF (PSF_net_) of the simulated sensor is first derived with nominal data acquisition parameters (altitude, speed, integration time, etc.). The PSF derivation accounts for the optical PSF (PSF_opt_), motion PSF (PSF_mot_) and detector PSF (PSF_det_) (excluding the dynamics of other factors such as platform and sensor vibration). Afterward, the derived PSF is convolved with the input HSI data. The output is then spatially degraded to the resolution of the simulated sensor with a nearest neighbor resampling technique. A MATLAB (Mathworks, Natick, MA, USA) implementation of the described workflow is provided in this manuscript. This MATLAB script can be used as-is or adapted as needed. In three practical example applications of the developed SR^2^ workflow, we show the importance of accounting for the sensor PSF when spatially resampling fine resolution HSI data for simulation. In the first example, the workflow is applied for HSI data cross-validation. This example shows the potential of using RPAS-HSI to bridge the gap between in situ spectroscopy data and coarser resolution HSI data. In the second example, the workflow is applied to aid in data acquisition planning. In this example, the simulation workflow establishes suitable scales and, by extension, data acquisition parameters for identifying a feature of interest. The final example application shows how the workflow can be implemented for data fusion between sensors that capture spectral information in different portions of the electromagnetic spectrum. This example generates a single full-range image from a VNIR and SWIR HSI dataset.

## Method workflow

**Fig. 1:**
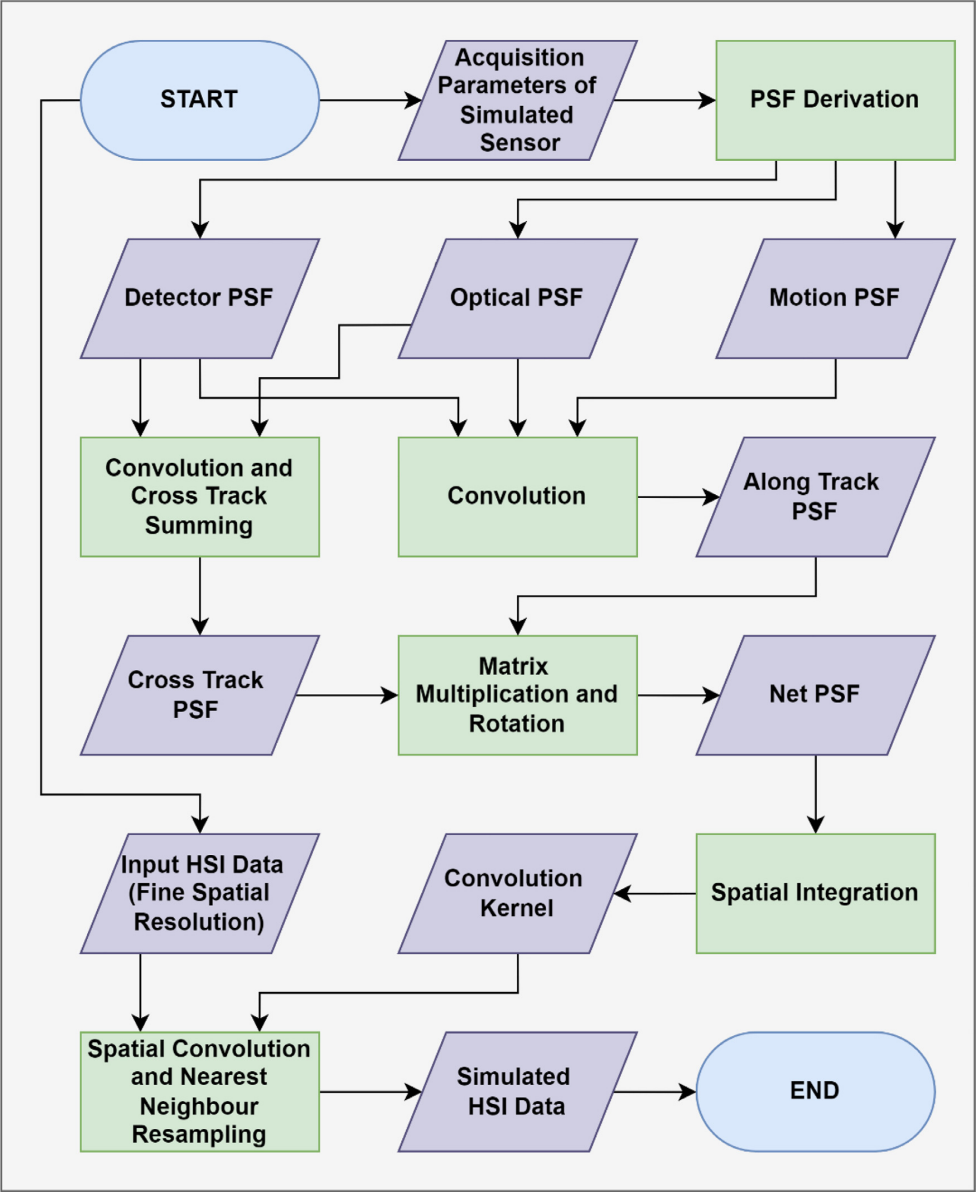
Flowchart of the Spatial Response Resampling (SR^2^) workflow. The workflow degrades fine spatial resolution hyperspectral imaging (HSI) data to the spatial characteristics of a coarser resolution sensor.

Following the methodology briefly outlined by Inamdar et al. [19], the SR^2^ simulation workflow derives the PSF_net_ of a specified sensor, convolving it with a finer resolution HSI dataset (Fig. 1). Before describing the SR^2^ workflow, it is necessary to define the terminology surrounding the spatial properties of HSI systems. In this work, the pixel resolution is defined by the full width at half maximum of the sensor PSF. The nominal pixel resolution is defined by the full width at half maximum of the scan PSF (convolution of the PSF_det_ and PSF_mot_), which is equal to the ground-projected instantaneous field of view (IFOV) (units of meters) in the cross track direction. Pixel size refers to the spatial dimensions of each square pixel in the geometrically corrected raster end product. With these definitions in mind, the workflow can be broken into three steps:

1. Derive PSF_net_ in two dimensions as a function of pixel displacement in the easting and northing directions.
2. Convolve PSF_net_ from step 1 with input HSI data.
3. Spatially resample input HSI data to the pixel size of the simulated sensor with a nearest neighbor resampling technique.

The PSF_net_ derivation in step 1 follows the calculations from [19], with a slight modification to account for cross track pixel summing. In this process, the PSF_opt_, PSF_mot_ and PSF_det_ are first derived. After, the cross track PSF_net_ is generated by convolving the PSF_opt_ and PSF_det_ while the along track PSF_net_ is obtained by convolving the PSF_opt_, PSF_mot_ and PSF_det_. In many data processing streams, adjacent cross track pixels are summed to boost signal levels and equalize spatial resolution between the cross track and along track directions [23]. In these scenarios, the cross track PSF_net_ must be modified to account for the degree to which data summing is applied. The PSF_net_ in the cross track direction at summing level s≥2|s∈Z can be defined by the following expression:

(1) $$PSF_{net,s}\left(x\right)=\left\{ \begin{array}{c} \sum_{k=1}^{k=\frac{s}{2}}PSF_{net}\left(x+\left(k-\frac{1}{2}\right)*rx\right)+PSF_{net}\left(x-\left(k-\frac{1}{2}\right)*rx\right),\;if\;s\geq 2|s\;\in 2Z\\ PSF_{net}\left(x\right)+\sum_{k=1}^{k=\frac{s-1}{2}}PSF_{net}\left(x+k*rx\right)+PSF_{net}\left(x-k*rx\right),\;otherwise \end{array}\right.$$

where *rx* is equal to the nominal pixel resolution in the cross track direction (width of the PSF_det_), x is the cross track displacement from the pixel center and $PSF_{net}\left(x\right)$ is the unsummed PSF_net_ in the cross track direction. The total PSF_net_ in two dimensions is then derived by vector multiplication of the PSF_net_ in the cross track (after data summing) and along track directions. To complete step 1, the total PSF_net_ is reparametrized as a function of pixel displacement in the easting and northing directions via matrix rotation by the nominal flight line heading (° True North). To generate the convolution kernel for step 2, the total PSF_net_ is spatially degraded to the same pixel size as the input HSI data. This is accomplished by spatially integrating the PSF_net_ in intervals equal to the pixel size of the input HSI data. The output matrix (referred to as the convolution kernel) is normalized to sum to unity. The kernel is then convolved with the input HSI data, blurring it based on the spatial characteristics of the simulated sensor. In the blurred HSI data, each pixel represents the average spectra that would contribute to a single pixel of the simulated sensor. It is important to note that the pixel size of the input HSI data does not change after convolution. To complete the simulation workflow, the blurred HSI data are spatially subset to the same pixel size as the simulated sensor using a nearest neighbor spatial resampling technique. A flowchart of the described workflow is shown in Fig. 1.

The presented workflow used a georeferenced raster data input. This was selected due to the popularity of the raster data model in optical remote sensing. This input was also effective for data handling and processing purposes as operations such as convolution can be carried out in a computationally efficient manner [24]. Although step 3 can be conducted in most commercially available geospatial software (e.g., ENVI's Resize function, ArcMap's (Esri, Redlands, CA, USA) Resample function), steps 1–2 may be challenging to implement for end users that are unfamiliar with PSFs and convolution. This work presents a MATLAB function (HSI_BLUR.m) that carries out steps 1–2 of the data simulation workflow to make the workflow more approachable for end users of all expertise levels.

The SR^2^ workflow relies on four implicit assumptions. Firstly, the derived PSF_net_ is assumed to be wavelength independent. In practice, PSFs are a function of wavelength. If a wavelength dependent PSF_opt_ is known for a sensor, this workflow can easily be modified accordingly. For the vast majority of end-users, this information is not easily accessible and thus the wavelength independence of the derived PSFs is necessary for simplification purposes. Secondly, the PSF_net_ assumes that the aircraft was flying at a constant speed in a direction that is perpendicular to the detector array. Without this assumption, the PSF_net_ would change across the imagery depending on small changes in speed and heading. Although a variable PSF_net_ would be more accurate, it imposes technical challenges in the convolution step and would slow down the workflow. Thirdly, the spatial response of the input data is assumed to be uniform over the spatial boundaries of each pixel. This assumption was necessary so that deconvolution was not required. The effect of this assumption is negligible so long as there is a large difference in scale between the input HSI data and the simulated output. Finally, the SR^2^ workflow assumes that the input HSI data is accurately georeferenced. Although this assumption has minimal implications for the SR^2^ workflow itself, it is important to consider when using an output from the workflow for tasks that rely on comparisons with external datasets. For instance, in the data fusion example, without ensuring that the VNIR and SWIR data are spatially aligned, their fusion could lead to an unrealistic spectrum.

## MATLAB function

The developed MATLAB function is based on the DHPC_DSM_BLUR.m function developed in Inamdar et al. [25]. The purpose of the DHPC_DSM_BLUR.m function was to convolve a digital surface model with the PSF of a coarser resolution HSI dataset for data fusion. Although not necessarily for data fusion, on a fundamental level, the SR^2^ workflow aims to accomplish the same task. However, instead of spatially degrading a DSM (1 band image), the workflow degrades an HSI dataset. Additional modifications have been made to the original DHPC_DSM_BLUR.m function to account for spatial summing in the cross track direction. As such, the PSF calculations are more robust and can simulate a wider array of HSI sensors.

The HSI_BLUR MATLAB function carries out four tasks: 1) derive hyperspectral PSF of the simulated sensor; 2) derive convolution kernel from PSF based on the characteristics of the input HSI data; 3) convolve input HSI dataset by convolution kernel, 4) output blurred imagery as ENVI standard data format. The HSI_BLUR.m function description defines the input and output parameters of the workflow.

The PSF derivation (task 1) follows the calculations presented in Inamdar et al. [19] using the implementation from Inamdar et al. [25]. In this process, the nominal pixel size of the simulated sensor (without considering pixel summing) is first calculated based on the field of view, number of cross track pixels, integration time and speed.

Next, the rectangular pulse detector PSF and gaussian optical PSF are calculated and convolved with one another to derive the net PSF in the cross track direction. It is important to note that, at this point, the net cross track PSF does not consider the dynamics of spatial pixel summing.

Afterward, the net cross track PSF is convolved with a derived rectangular pulse motion PSF. The result of this convolution is the net PSF in the along track direction.

Next, the cross track net PSF is spatially summed as per the setup of the simulated sensor.

The net PSF in 2-dimensions is derived through vector multiplication of the cross track and along track net PSFs. The rows and columns of the resultant matrix correspond with the along track and cross track displacement (in meters) from the center of the pixel. Since the rows and columns of the input HSI data correspond to the northing and easting directions, respectively, the net PSF must be rotated by the flight line heading of the simulated sensor before convolution. After rotation, task 1 is completed.

To complete task 2, the input HSI data must first be imported, and the pixel size must be extracted.

To generate the convolution kernel and complete task 2, the rotated net PSF is spatially integrated in intervals equal to the input HSI dataset pixel size and normalized to sum to unity.

Afterward, the input HSI dataset is convolved with the derived kernel, blurring the imagery based on the PSF of the simulated sensor (completing task 3).

Task 4 is completed by saving the blurred HSI dataset to a new ENVI standard file [26] in the same location as the input HSI dataset. This new file is named after the input HSI dataset, appended with “_conv”.

Below, we provide an example MATLAB code that generates a blurred HSI dataset by calling HSI_BLUR.m.

It is important to note that the provided MATLAB function does not apply the final nearest neighbor spatial resampling stage of the workflow. As previously mentioned, this can be done in most commercially available image analysis software (e.g., the Resize function from ENVI or the Resample function in ArcMap).

## Example applications of the spatial response resampling workflow

In this work, we give three practical example applications of the SR^2^ workflow to show the importance of accounting for the sensor PSF when spatially resampling HSI data to simulate the spatial characteristics of a coarser resolution sensor. These applications use HSI data collected from two field sites: The Mer Bleue Peatland (MBP) in Ontario, Canada and the Puerto Jiménez Airport (PJA) in Puntarenas, Costa Rica. The MBP is a ∼8500-year-old ombrotrophic bog characterized by a hummock-hollow microtopography consisting of a base layer of peat and patches of vascular plants (i.e., hummock) [27]. The MBP has been studied extensively using airborne hyperspectral remote sensing. For instance, HSI data from the MBP has been leveraged to study water table depth, net ecosystem exchange [28], maximum gross photosynthesis, gravimetric water content and CO_2_ uptake efficiency [29]. The PJA is located on the Osa peninsula and contains many urban features (e.g., roads and buildings). The PJA was a validation site for the Mission Airborne Carbon 13 (MAC-13) project, an initiative to derive aboveground biomass/carbon estimates in five highly diverse ecosystems in Costa Rica [30].

**Fig. 2:**
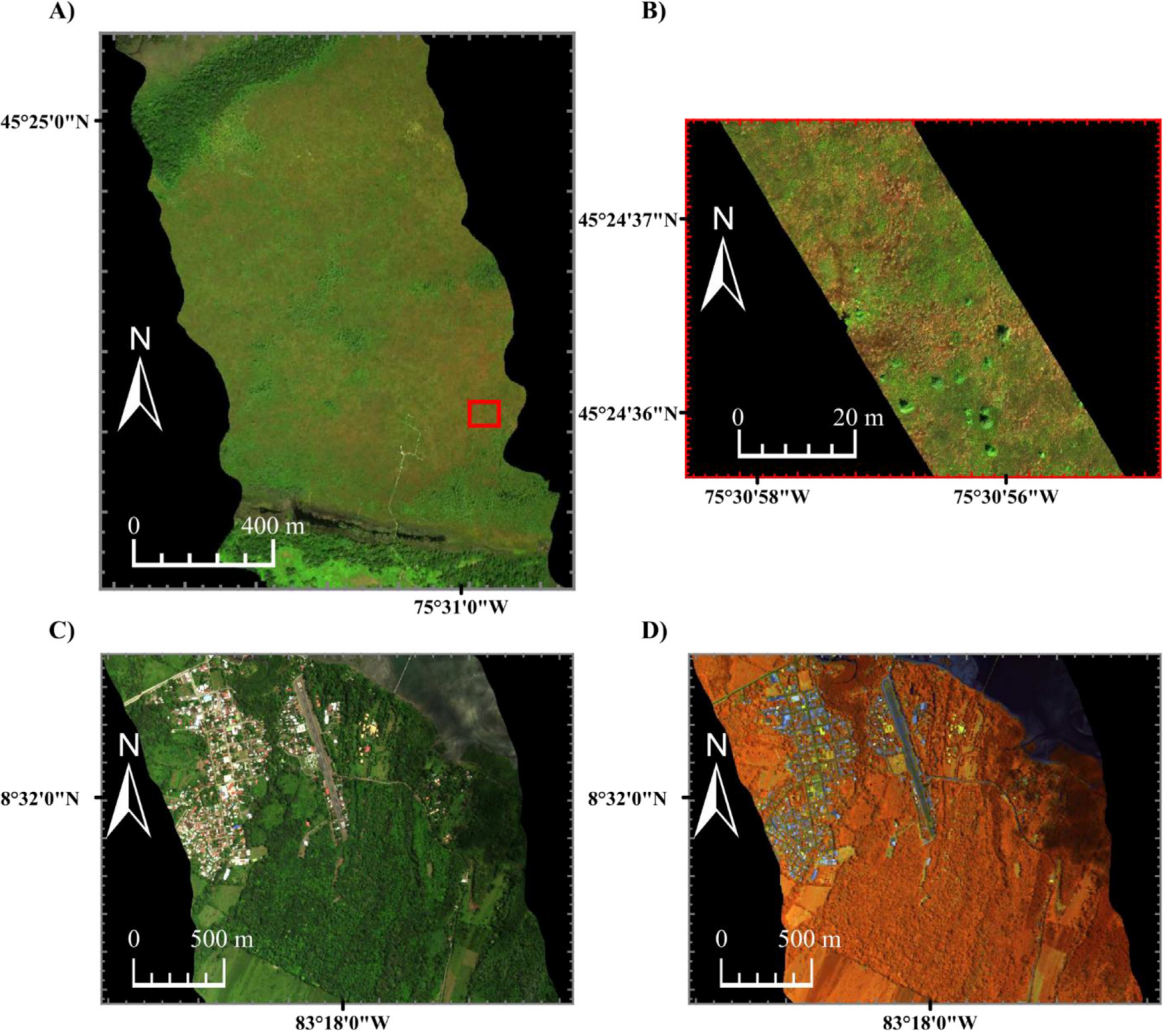
The hyperspectral imaging data used to show the utility of the developed Spatial Response Resampling (SR^2^) workflow. CASI-1500 and µCASI-1920 data were collected over the Mer Bleue Peatland (MBP), while CASI-1500 and SASI-640 data were collected over the Puerto Jiménez Airport (PJA). A) Hyperspectral imaging data collected over the MBP with the CASI-1500 (*R* = 640.8 nm, *G* = 549.9 nm, *B* = 459.0 nm, linearly scaled from 0% to 12%). The red box shows the location where the µCASI-1920 data was collected. B) Hyperspectral imaging data collected over the MBP with the µCASI-1920 (*R* = 639.6 nm, *G* = 550.3 nm, *B* = 459.0 nm, linearly scaled from 0% to 12%). C) Hyperspectral imaging data collected over the PJA with the CASI-1500 (*R* = 641.2 nm, *G* = 550.3 nm, *B* = 458.3 nm, linearly scaled from 0% to 20%). D) Hyperspectral imaging data collected over the PJA with the SASI-640 (*R* = 1240.1 nm and linearly scaled from 0% to 60%, *G* = 1540.7 nm and linearly scaled from 0% to 50%, *B* = 1846.0 nm and linearly scaled from 0% to 30%).

HSI data were collected using three pushbroom hyperspectral imagers: the micro-Compact Airborne Spectrographic Imager (µCASI-1920, ITRES, Calgary, AB, Canada), the Compact Airborne Spectrographic Imager (CASI-1500, ITRES, Calgary, AB, Canada) and the Shortwave Airborne Spectrographic Imager (SASI-640, ITRES, Calgary, AB, Canada). The µCASI-1920 and the CASI-1500 collect spectral information in the VNIR from 450 to 900 nm, while the SASI-640 collects spectral information in the SWIR from 900 to 1900 nm. Because the µCASI-1920 was mounted on a DJI Matrice 600 Pro-RPAS, it is capable of collecting finer spatial resolution data (< 5 cm) [3] than the CASI-1500 and SASI-640 sensors, which are mounted in a Twin Otter fixed-wing manned aircraft. This study specifically analyzes µCASI-1920 and CASI-1500 data collected from the MBP in addition to CASI-1500 and SASI-640 data from the PJA (see Fig. 2). The data acquisition parameters and sensor properties associated with each dataset are given in Table 1. Before application, all HSI data were radiometrically corrected, atmospherically compensated and geometrically corrected using software developed by the sensor manufacturer and ATCOR4 (ReSe, Wil, Switzerland), as described by Soffer et al. [31] and Osei Darko et al. [32].

**Table 1:** Parameters for the hyperspectral imaging data acquired over the Mer Bleue Peatland (MBP) and Puerto Jiménez Airport (PJA) with the SASI-644, CASI-1500 and µCASI-1920.

Parameter	MBP (µCASI-1920)	MBP (CASI-1500)	PJA (CASI-1500)	PJA (SASI-640)
Date (dd-mm-yyyy)	15-07-2019	15-07-2019	29-04-2013	29-04-2013
Image start time (hh.mm.ss GMT)	15.44.49	15.44.38	15.07.55	15.07.55
Total Number of Cross Track Pixels	1920	1500	1500	644
Effective Number of Cross Track Pixels	1833	1496	1493	640
Sensor Field of View (°)	34.21	39.9	39.9	39.7
Nominal Flight Line Heading (° True North)	156	341	343	343
Nominal Altitude (m | ft)	45 | 148	1133 | 3717	2586 | 8484	2586 | 8484
Nominal Speed (m/s | kn)	2.7 | 5.2	41.6 | 80.9	61.7 | 120.0	61.7 | 120.0
Integration Time (ms)	9	48	32	4.1
Frame Time (ms)	11	48	32	16.7
Full width at half maximum of Optical Point Spread Function (pixels)	1.1	1.1	1.1	1.1
Cross Track Summing (pixel)	1	2	1	1
Nominal Cross Track Pixel Resolution (m)	0.03	1.10	1.25	2.92
Nominal Along Track Pixel Resolution (m)	0.03	1.97	1.99	2.92
Pixel Size of Georeferenced Raster (m)	0.03	1.0	1.25	3.5

The first example application shows the utility of the SR^2^ workflow for data cross-validation. Specifically, the CASI-1500 imagery from the MBP was cross-validated using the μCASI-1920 data. In the second example application, the SR^2^ workflow was applied for flight planning. In this example, the simulation workflow used the µCASI-1920 data to establish appropriate CASI-1500 data acquisition parameters for identifying hummocks and hollows within the MBP. The final example application shows how the SR^2^ workflow can be implemented for data fusion between sensors that capture spectral information in different portions of the electromagnetic spectrum. In this application, the CASI-1500 data from the PJA was fused with the SASI-640 data to generate a spatially coherent full-range spectrum.

## Data cross-validation application

In the remote sensing literature, cross-validation is a process whereby a measurement with known uncertainty is used to assess the accuracy of an independent measurement. Typically, *in situ* data collected at the ground level is used to cross-validate measurements collected at the airborne level, which can then be used to cross-validate spaceborne measurements. Cross-validation of *in situ* and imaging spectroscopy data at the airborne and spaceborne levels requires a detailed understanding of the measurement process, the involved spatial-spectral scales and the processing applied to the data [33]. Depending on the sampling strategy and the characteristics of the target (size, spectral variability), it can be difficult to acquire *in situ* data that is spatially coherent with airborne imaging spectroscopy data. Additional problems arise in cross-validation efforts as it is difficult to collect *in situ* data that samples materials across the spatial extent covered by airborne sensors. RPAS-HSI data presents a potential solution to bridge the gap between airborne HSI data and *in situ* data via cross-validation. This study analyzes the utility of the SR^2^ workflow in cross-validating higher altitude airborne HSI data with finer resolution RPAS-HSI data.

**Fig. 3:**
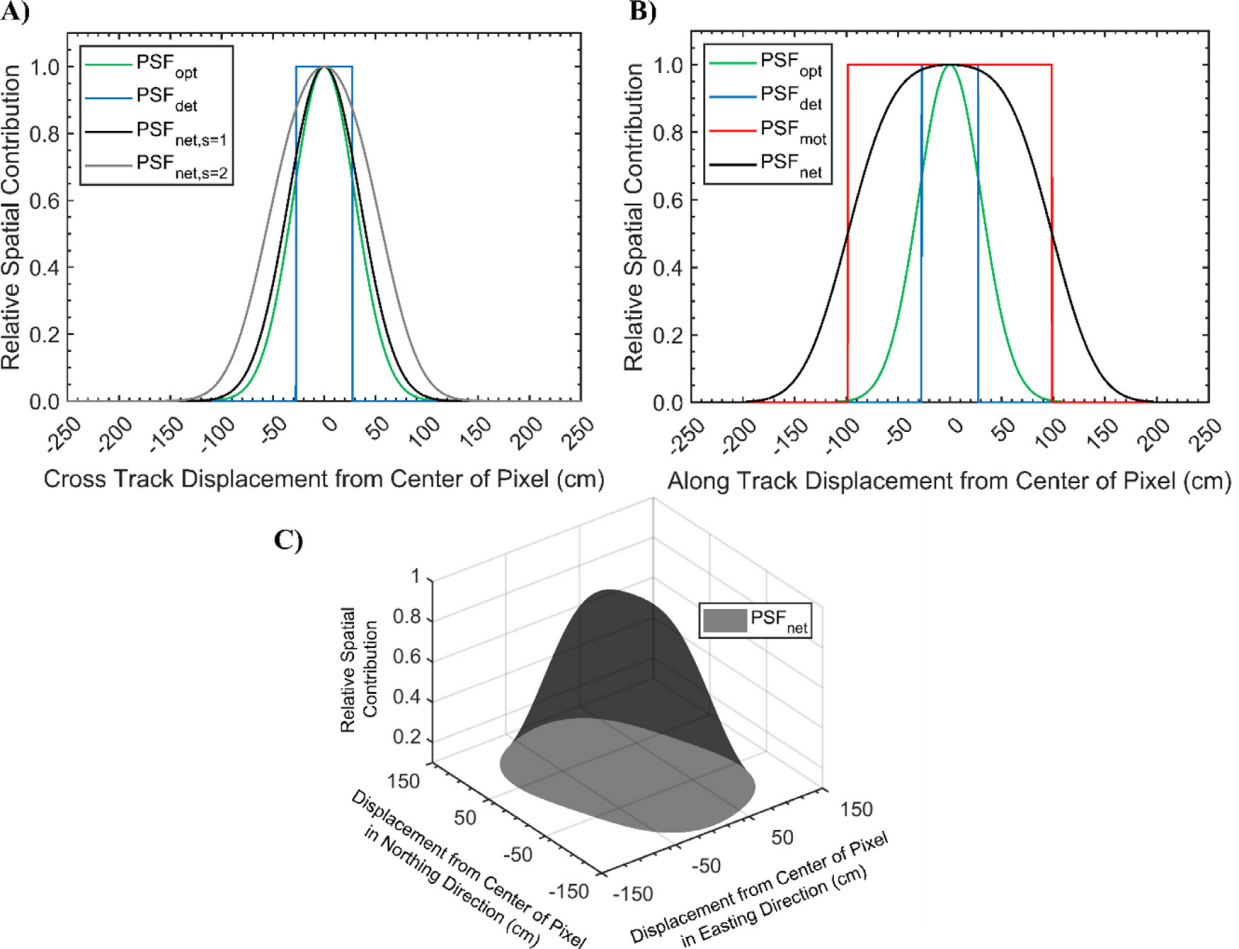
The point spread function (PSF) for the CASI-1500 data collected over the Mer Bleue Peatland. A) The optical PSF (PSF_opt_), detector PSF (PSF_det_) and net PSF (PSF_net_) in the cross track direction (unsummed and summed PSFs are designated by the *s* = 1 and *s* = 2 tags, respectively). B) The PSF_opt_, PSF_det_, motion PSF (PSF_mot_) and PSF_net_ in the long track direction. C) The PSF_net_ as a function of displacement from the center of the pixel in the easting and northing directions. The grid in the x-y plane corresponds to the pixel size in the final georeferenced end product. When studying the PSF_net_ in panel C, less than 37% of the signal originates within the square spatial boundaries defined by pixel size in the final data end product.

The μCASI-1920 dataset was input to the SR^2^ workflow to simulate the MBP CASI-1500 data with the flight parameters in Table 1. The PSF associated with the MBP CASI-1500 data can be seen in Fig. 3. The simulated image was compared to a conventional data simulation approach where the μCASI-1920 imagery was spatially degraded using a pixel aggregate averaging approach. In this comparison, the mean and standard deviation of each spectral band were calculated for the two degraded μCASI-1920 datasets and the original CASI-1500 dataset. For comparability, the CASI-1500 data were spatially subset to the area covered by the μCASI-1920 imagery before calculating the mean and standard deviation of each spectral band.

**Fig. 4:**
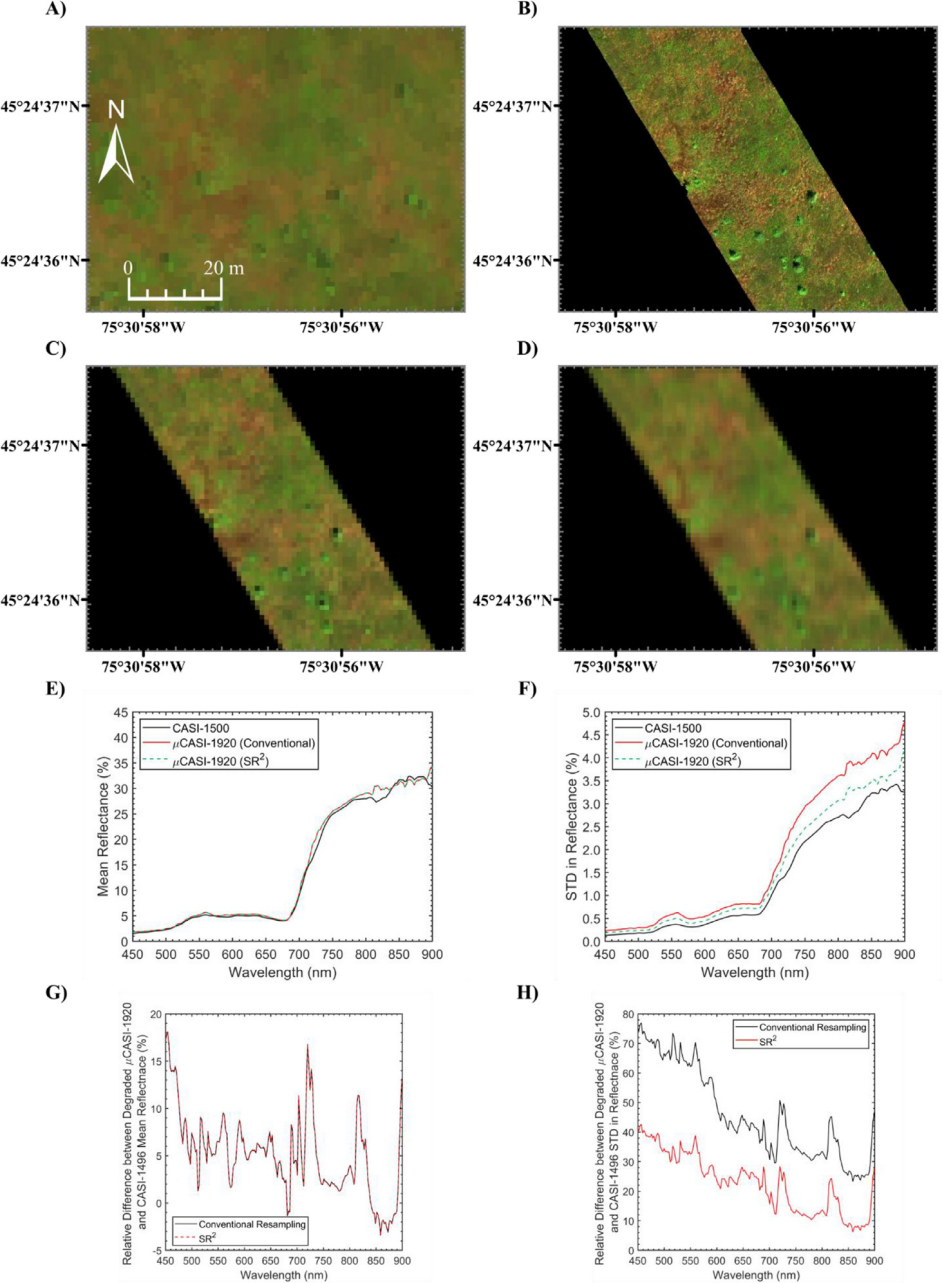
Example data cross-validation application of the Spatial Response Resampling (SR^2^) workflow. A) Spatial subset of the Mer Bleue Peatland (MBP) CASI-1500 hyperspectral imaging data (*R* = 640.8 nm, *G* = 549.9 nm, *B* = 459.0 nm, linearly scaled from 0% to 12%). B) Original µCASI-1920 hyperspectral imaging data collected over the MBP (*R* = 639.6 nm, *G* = 550.3 nm, *B* = 459.0 nm, linearly scaled from 0% to 12%). C) Spatially degraded µCASI-1920 hyperspectral imaging data (*R* = 639.6 nm, *G* = 550.3 nm, *B* = 459.0 nm, linearly scaled from 0% to 12%) generated using conventional resampling methodologies (pixel aggregate method). D) Spatially degraded µCASI-1920 hyperspectral imaging data (*R* = 639.6 nm, *G* = 550.3 nm, *B* = 459.0 nm, linearly scaled from 0% to 12%) generated using the SR^2^ workflow. E) The mean of each spectral band from the two spatially degraded µCASI-1920 images and the original CASI-1500 imagery, spatially subset to cover the same extent. F) The standard deviation in each spectral band from the two spatially degraded µCASI-1920 images and the CASI-1500 imagery, spatially subset to cover the same extent. G) Relative difference in the calculated mean reflectance spectrum between the original CASI-1500 imagery and the two spatially degraded µCASI-1920 images. H) Relative difference between the calculated standard deviation in reflectance of the original CASI-1500 imagery and the two spatially degraded µCASI-1920 images. The simulated data product generated using the SR^2^ workflow was the most spatially consistent with the CASI-1500 imagery. The mean in the reflectance was consistent between the two simulated data products and the CASI-1500 data. The standard deviation calculated for the simulated imagery derived from the SR^2^ workflow was the closest to that of the CASI-1500 imagery, indicating that the datasets are characterized by similar levels of sensor blurring.

Fig. 4A-D shows the original CASI-1500 and μCASI-1920 imagery, in addition to the two simulated data products. The mean reflectance spectra (Fig. 4E) of the two simulated data products were consistent with the mean spectrum of the CASI-1500 imagery. The standard deviation (Fig. 4F) in the reflectance spectra of the conventional data simulation end product was 45.86% larger on average than the standard deviation measured from the original CASI-1500 data (ranging from 23.37% to 76.91%) (see Fig. 4H). The standard deviation in the reflectance spectra of the SR^2^ data simulation end product was only 22.65% larger on average than the standard deviation measured from the CASI-1500 data (ranging from 6.29% to 42.62%) (see Fig. 4H). Inamdar et al. [19] show that the overlap in the sensor PSF of adjacent pixels results in spatial autocorrelation that changes observed spectral variance. If the spatial properties of the degraded μCASI-1920 imagery are perfectly consistent with that of the CASI-1500, then the standard deviation in the reflectance spectrum should be identical. It is important to recognize that the standard deviation values calculated for the original and simulated CASI-1500 data will never be identical due to factors such as jitter, increased noise levels, viewing geometries and other sensor-related phenomena, in addition to intrinsic properties of the observed matter such as material bidirectional reflectance distribution functions. However, the observed reduction in the standard deviation implies that the simulated dataset output from the SR^2^ workflow was more spatially consistent with the CASI-1500 data than the conventional data product. If the conventional data simulation product was used in data cross-validation efforts, the additional variation in the reflectance spectra could contribute to the overall errors. The additional errors would unnecessarily increase the overall uncertainty in the CASI-1500 data during cross-validation efforts. Overall, the SR^2^ workflow ensured that all data used in cross-validation efforts were spatially consistent for data validation efforts.

## Flight planning application

HSI acquisition is monetarily expensive, requiring considerable effort from experts during flight planning and data acquisition [3]. As a result, it is practically infeasible to test multiple data acquisition parameters to determine how to optimally collect data for a specific scientific question. The SR^2^ workflow presents a solution to this problem, showing an example where fine resolution HSI data can be used to identify optimal data acquisition parameters to collect coarser resolution HSI data capable of identifying a user-defined target with minimal mixing. In the flight planning example application, the SR^2^ workflow used the µCASI-1920 data to establish appropriate CASI-1500 data acquisition parameters for identifying hummock and hollow microforms within the MBP. A hollow microform is at or below the water table in the peatland and is primarily composed of exposed mosses (e.g., *Sphagnum* spp.), while a hummock microform is an elevated mound in the peatland surface where vascular plants densely cover the underlying mosses [34,35]. In the MBP, hummocks and hollows differ in absolute elevation by as much as 0.30 m and are separated by an approximate horizontal distance of 1–2 m [36,37]. In peatlands, hummock-hollow microtopography provides diversity in ecohydrological structure and biogeochemical function that is integral to the negative feedbacks that maintain the long-term stability of peatland carbon [34,[37], [38], [39]. As such, characterization of hummock-hollow microtopography is critical to understanding and modeling complex hydrological and biogeochemical processes, in addition to validating satellite-derived products such as water table depth and net ecosystem exchange [28,^40^,41].

**Fig. 5:**
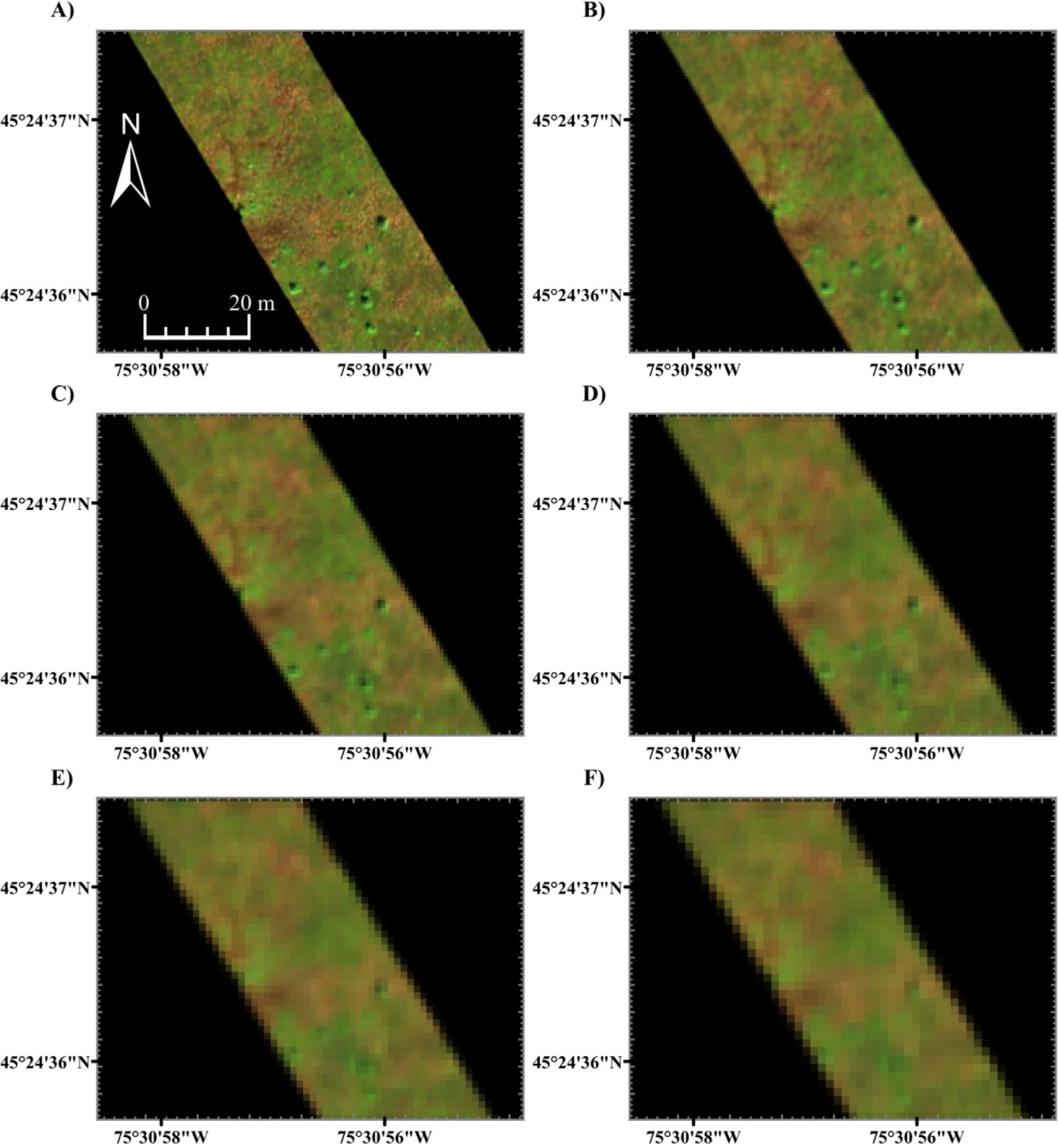
Example flight planning application of the Spatial Response Resampling (SR^2^) workflow. A-F) Spatially degraded µCASI-1920 hyperspectral imaging data (*R* = 639.6 nm, *G* = 550.3 nm, *B* = 459.0 nm, linearly scaled from 0% to 12%) generated using the SR^2^ workflow with the data acquisition parameters in Table 2. Panels A-F correspond with simulations of the scene at scale 1 (0.25 m), scale 2 (0.50 m), scale 3 (0.75 m), scale 4 (1.0 m), scale 5 (1.25 m) and scale 6 (1.5 m), respectively. In general, the hummocks appear green in color while hollows appear red. Users can analyze these datasets to understand the required spatial resolution for their particular application. In this case, the microtopography of the Mer Bleue peatland becomes less observable at coarser resolutions.

In the flight planning data application, the µCASI-1920 imagery was spatially degraded using the SR^2^ workflow based on the characteristics of the CASI-1500 sensor with various data acquisition parameters (see Table 2). Each set of data acquisition parameters was selected to simulate data that the CASI-1500 could potentially acquire at various nominal spatial resolutions (0.25 m, 0.5 m, 0.75 m, 1.00 m, 1.25 m, 1.50 m). Fig. 5 shows the spatially degraded µCASI-1920 datasets that simulated various flight configurations of the CASI-1500. At 25 cm, the hummock-hollow microtopography could be clearly observed. As progressively coarser resolution CASI-1500 images were simulated, the hummock-hollow microtopography was more difficult to identify qualitatively.

**Table 2:** Tested CASI-1500 data acquisition parameters for degrading the µCASI-1920 at multiple spatial scales. The different spatial scales were acquired by modifying sensor altitude and integration time. The bolded entries indicate the parameters that were varied between scales. It is important to note that it is not feasible to maintain such precise altitudes over long durations at the airborne level with manned aircrafts.

Parameter	Scale 1	Scale 2	Scale 3	Scale 4	Scale 5	Scale 6
Number of Cross Track Pixels	1500	1500	1500	1500	1500	1500
Sensor Field of View (°)	39.86	39.86	39.86	39.86	39.86	39.86
Nominal Flight Line Heading (° True North)	0	0	0	0	0	0
Nominal Altitude (m | ft)	**517** | **1696**	**1034** | **3392**	**1551** | **5088**	**2068** | **6785**	**2575** | **8448**	**3092** | **10,144**
Nominal Speed (m/s | kn)	41 | 80	41 | 80	41 | 80	41 | 80	41 | 80	41.15 | 80
Integration Time (ms)	**6**	**12**	**18**	**24**	**30**	**36**
Full width at half maximum of Optical Point Spread Function (pixels)	1.1	1.1	1.1	1.1	1.1	1.1
Cross Track Summing (pixel)	1	1	1	1	1	1
Swath (m)	375	750	1125	1500	1867	2242
Nominal Pixel Resolution (m)	0.25	0.5	0.75	1.0	1.25	1.50

**Fig. 6:**
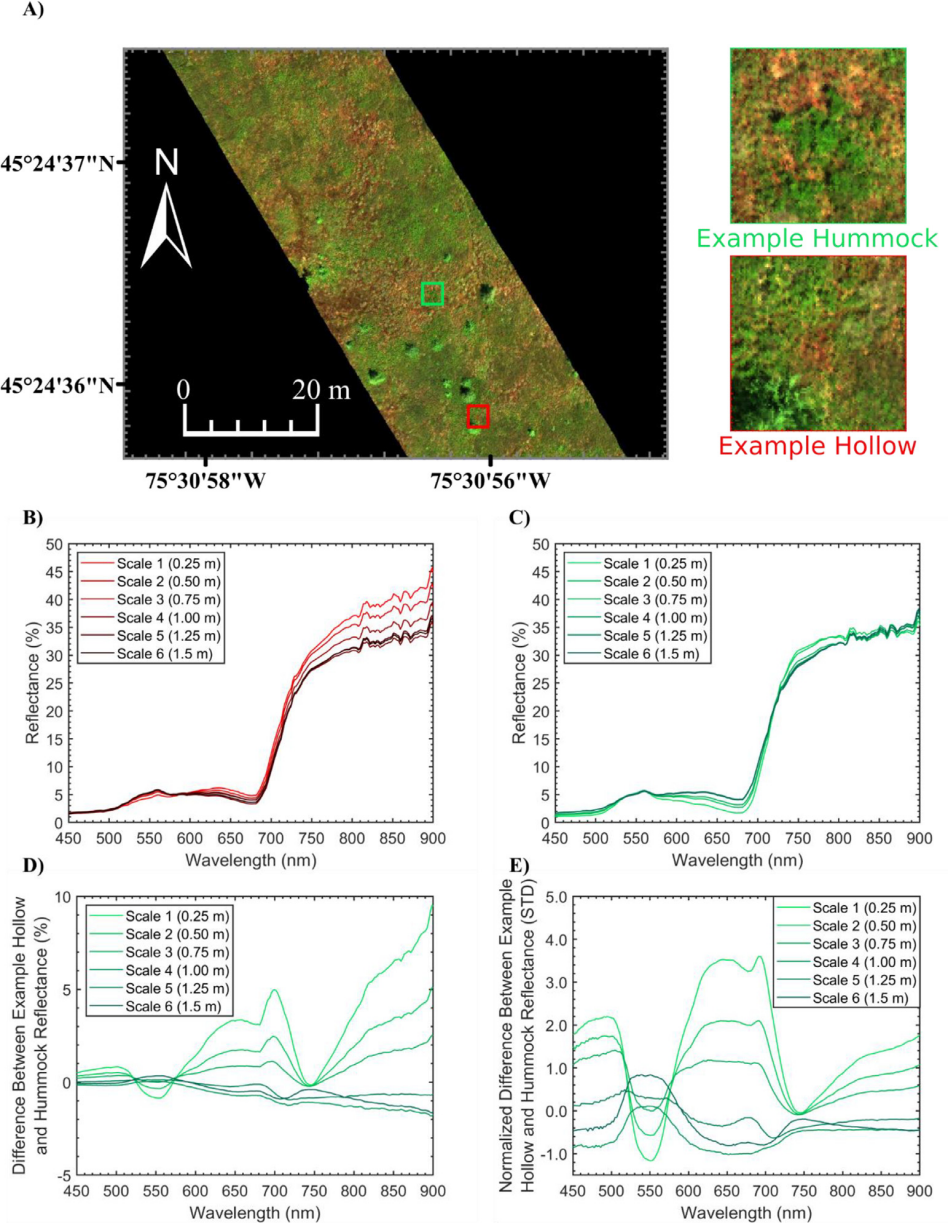
Hummock and hollow spectra extracted from the spatially degraded µCASI-1920 hyperspectral imaging data generated using the SR^2^ workflow with the data acquisition parameters in Table 2. A) Original µCASI-1920 hyperspectral imaging data (*R* = 639.6 nm, *G* = 550.3 nm, *B* = 459.0 nm, linearly scaled from 0% to 12%). The analyzed example hummock and hollow were identified in the image. In general, the hummocks appear green in color while hollows appear red. Panels B and C show the example hollow and hummock spectra, respectively. Panel D shows the difference between the hollow and hummock spectra at different spatial scales. Panel E shows the normalized difference between the hollow and hummock spectra at different spatial scales in units of standard deviation (STD). These values were obtained by dividing the difference spectrum in panel D by the standard deviation in each spectral band of the image from which the spectra were obtained. The absolute value of the normalized difference is representative of separability between the example hummock and hollow as a function of wavelength. As the spatial scale becomes coarser, hummock and hollow reflectance spectra become more similar due to mixing with neighboring endmembers. For instance, at fine spatial resolutions, the sphagnum from the hollow spectra can be observed by the shifted green peak and the high NIR reflectance. At scale 3–6, these characteristics were lost, and the spectra were more consistent with the example hummock.

The detectability of hummocks and hollows was analyzed by extracting spectra from a small (∼1 m) example hummock and hollow at the MBP (see Fig. 6A). Hollows are mainly composed of exposed *Sphagnum* mosses. As such, the spectral properties of hollows differ from hummocks [42,43], which are composed of *Sphagnum* mosses densely covered by vascular plants. The predominant difference between *Sphagnum* moss and vascular plant reflectance is in the location and magnitude of the green peak, the red edge inflection point and the magnitude of reflectance in the near infrared [42], [43], [44]. This is due to differences in pigmentation and cell and canopy structure [42], [43], [44]. Fig. 6B-C shows the spectra extracted from the example hollow and hummock within each simulated scene. The difference between the hollow and hummock spectra at each scale was displayed in Fig. 6D. To measure separability, the difference spectrum at each scale was normalized by the standard deviation in each band of the utilized HSI data. The absolute value of the normalized difference is representative of separability between the example hummock and hollow as a function of wavelength in units of standard deviations (STD) (see Fig. 6E).

At fine spatial resolutions, mixing in the hummock and hollow spectra was less prominent. For instance, the hollow spectrum at scale 1 (see Fig. 6B) was representative of *Sphagnum* mosses such as *Sphagnum divinum*, with a notable shift in the green peak towards longer wavelengths and high reflectance in the near infrared likely due to low near-surface moisture content. Similarly, the hummock spectrum at scale 1 was representative of vascular plants, with a red absorption feature from ∼650 to 680 nm (see Fig. 6C) that was not observable in the hollow spectrum. As the resolution became coarser, mixing between hummocks and hollows was more prominent and the difference between the example spectrum from each microform decreased (Fig. 6D-E). For example, over the spectral range of the red absorption feature typically observed in vascular plants (∼650–680 nm), the difference in reflectance between the example hollow and hummock spectrum was 3.24% on average at scale 1, ranging from 3.10% to 3.36%. The corresponding normalized difference was equal to 3.40 STD on average, ranging from 3.25 to 3.52 STD. At scale 6, the difference between the hummock and hollow spectrum over the same spectral range was only 0.49% on average, ranging from 0.47% to 0.52%. The corresponding normalized difference was equal to 0.77 STD on average, ranging from 0.73 −0.81 STD. This practically implies that hummocks and hollows were more than 4 times as separable at scale 1 when compared to scale 5 over the 680–700 nm spectral range. Given the generally low separability of hummocks and hollows at scales 3–6 (normalized difference between hummock and hollow spectrum < 1.36 STD, see Fig. 6E), the flight parameters from scales 1–2 were the most suitable for aerial campaigns interested in the microtopography at the MBP.

When selecting flight parameters, it is critical to consider logistical constraints. For instance, although an integration time of 6 ms may be technically possible, it would require on-chip summing in the spectral domain which may result in suboptimal data applications. Similarly, high altitudes may not be practically feasible. Overall, the simulation workflow is useful in determining the data acquisition parameters necessary to detect features of interest.

## Data fusion of VNIR and SWIR imagery

Inamdar et al. [45] developed a data fusion approach that synergistically integrated surface elevation data into HSI data. In their work, a fine spatial resolution digital surface model was convolved with the PSF of a coarser resolution HSI dataset to make the elevation and spectral data more spatially consistent. Following the same logic, the SR^2^ workflow can be used for data fusion between sensors that capture spectral information in different portions of the electromagnetic spectrum (e.g., visible near infrared (VNIR) and shortwave infrared (SWIR)) at different spatial scales. In this example application, the CASI-1500 and SASI-640 data collected over the PJA were fused to generate a full-range (450 nm to 1900 nm) image.

Due to differences in sensor characteristics, the nominal spatial resolution of the SASI-640 imagery (2.92 m in cross and along track) was coarser than that of the CASI-1920 imagery (1.25 m and 1.99 m in cross and along track, respectively). To ensure that the reflectance spectrum was spatially coherent between the VNIR and SWIR during data fusion, the CASI-1920 imagery needed to be spatially degraded. As such, the CASI-1500 data was input to the data simulation workflow and spatially degraded based on the spatial response of the SASI-640 derived from the flight parameters in Table 1. Before generating a single full-range data product from the blurred CASI-1500 and unmodified SASI-640 data, the CASI-1500 data was spatially aligned to the SASI-640 data via the 1st order polynomial image-to-image registration technique in ENVI. This spatial alignment was necessary to reduce spatial offsets between the CASI-1920 and SASI-640 data. To generate a full-range data product after the simulation workflow and geo-alignment, the spatially degraded CASI-1500 image and unmodified SASI-640 image were stacked via ENVI using a nearest neighbor resampling technique. To showcase the utility of the SR^2^ workflow in data fusion, the derived full-range data product was compared against a conventional full-range data product generated by stacking the geoaligned CASI-1500 data and the SASI-640 data using a nearest neighbor resampling technique in ENVI. The two data products were initially evaluated by observing the mean and standard deviation in the reflectance spectrum from two 280 m × 280 m (80 × 80 pixels) regions of interest (forest and urban) in the fused data products.

**Fig. 7:**
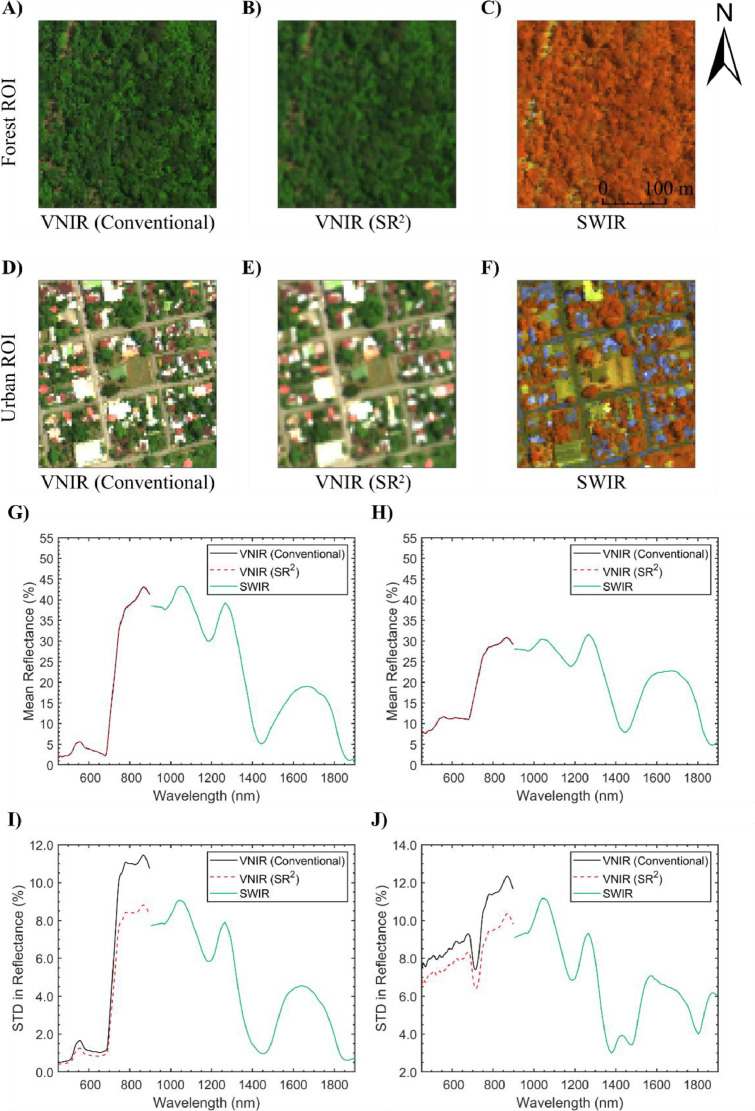
Example data fusion application of the spatial response resampling (SR^2^) workflow for generating a full-range image from separate VNIR and SWIR hyperspectral imagery. The two tested full-range data products were the same in the shortwave infrared (SWIR) and only distinguishable in the visible near infrared (VNIR). The conventional full-range data product was generated using a nearest neighbor resampling technique, while the novel data fusion approach used the SR^2^ technique described in this study. A-C) the forest region of interest in the VNIR (*R* = 641.2 nm, *G* = 550.3 nm, *B* = 458.3 nm, linearly scaled from 0% to 20%) and the SWIR (*R* = 1240.1 nm and linearly scaled from 0% to 60%, *G* = 1540.7 nm and linearly scaled from 0% to 50%, *B* = 1846.0 nm and linearly scaled from 0% to 30%) for both full-range data products. d-F) The urban region of interest in the VNIR and SWIR (RGB display identical to the forest region of interest) for both full-range products. G) The mean reflectance spectrum from the conventional and SR^2^ full-range end products over the forest region of interest. H) The mean reflectance spectrum from the conventional and SR^2^ full-range end products over the urban region of interest. Subplots I and J display the standard deviation in the reflectance spectrum shown in subplots G and H, respectively. The offset between the VNIR and SWIR in subplots I and J shows that the SR^2^ workflow is critical in ensuring that the merged SASI-640 imagery and CASI-1500 imagery are spatially consistent.

**Fig. 8:**
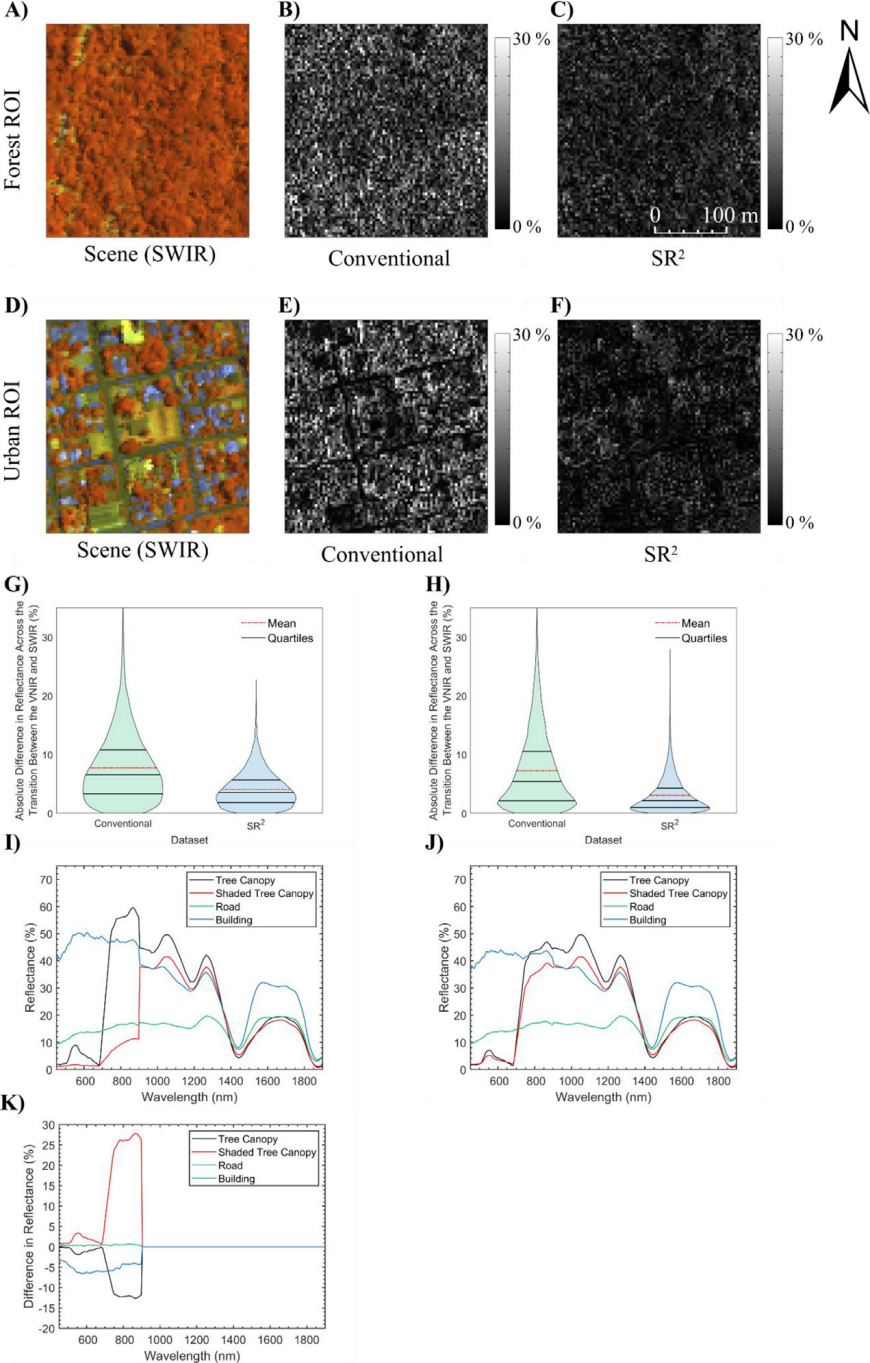
Example data fusion application of the spatial response resampling (SR^2^) workflow for generating a full-range image from separate VNIR and SWIR hyperspectral imagery. The two tested full-range data products were the same in the shortwave infrared (SWIR) and only distinguishable in the visible near infrared (VNIR). The conventional full-range data product was generated using a nearest neighbor resampling technique, while the novel data fusion approach used the SR^2^ technique described in this study. A) The forest region of interest (ROI) in the SWIR (*R* = 1240.1 nm and linearly scaled from 0% to 60%, *G* = 1540.7 nm and linearly scaled from 0% to 50%, *B* = 1846.0 nm and linearly scaled from 0% to 30%). Panels B and C show the absolute difference in reflectance across the transition between the VNIR and SWIR for the two studied full-range data products in the forest ROI. D) The urban ROI in the SWIR (displayed identically to panel A). Panels E and F show the absolute difference in reflectance across the transition between the VNIR and SWIR for the two studied full-range data products in the urban ROI. G) Violin plot (includes mean and quartiles) of the absolute difference in reflectance across the transition between the VNIR and SWIR for the two studied full-range data products in the forest ROI. H) Violin plot (includes mean and quartiles) of the absolute difference in reflectance across the transition between the VNIR and SWIR for the two studied full-range data products in the urban ROI. I) Spectra from various materials extracted from the conventional data fusion end product within the studied ROIs. J) Spectra from various materials extracted from the SR^2^ data fusion end product within the studied ROIs. The offset between the VNIR and SWIR in the conventional full-range data product would leave the spectra unusable in spectroscopy analyses such as material identification and characterization. K) Difference between panels I) and J).

Fig. 7A-F shows the studied ROIs from the two generated full-range products in the VNIR and SWIR. As seen in Fig. 7G-H, the difference between the mean VNIR reflectance spectra in the conventional and SR^2^ data products was marginal (< 0.12%). This was expected as convolution theoretically has no effects on first-order statistics such as mean [19] over large enough regions. Fig. 7I-J shows that the conventional full-range data product had a larger standard deviation than the SR^2^ workflow derivative. The offset in standard deviation between the VNIR and SWIR was larger in the conventional full-range product (3.02% and 2.58% for the forest and urban ROIs, respectively) than in the SR^2^ derivative (0.58% and 0.28% for the forest and urban ROIs, respectively). Based on the results previously discussed by Inamdar et al. [19], this implies that the spatial properties of the CASI-1500 data are more consistent with that of the SASI-640 when using the SR^2^ workflow. To expand on this analysis, the absolute offset in reflectance spectra between the VNIR and SWIR for the two studied full-range data products was calculated on a pixel-by-pixel basis (see Fig. 8) over the studied ROIs. The VNIR reflectance spectra derived from the SR^2^ workflow were more consistent with the SWIR data. As seen in the violin plots from Fig. 8G-H, the mean absolute difference in reflectance across the transition between the VNIR and SWIR in the SR^2^ full-range end product was 4.08% and 3.06% for the forest and urban ROIs, respectively. These values were much smaller than those recorded for the conventional full-range product, which was 7.72% and 7.25% for the forest and urban ROIs, respectively. Visual inspection of the violin plots also reveals that the distribution of the offset between the VNIR and SWIR portions skews closer to zero for the SR^2^ data derivative than the conventional end product.

To gain some insight into the cause of the VNIR-SWIR offset, Fig. 8I-J shows spectra of various features (tree canopy, shaded tree canopy, building and road) within the two example ROIs. Fig. 8K shows the difference in reflectance between Fig. 8J and Fig. 8I. In the conventional full-range product, the spectra were inconsistent between the VNIR and SWIR (see Fig. 8I). For instance, in the shaded tree canopy spectra, the VNIR spectra appeared to be from a shaded canopy exclusively, while the SWIR spectra appeared to be a mixture of the shaded canopy and the unshaded surrounding materials. The discrepancy in the material composition of the shaded canopy spectrum resulted in a large offset of 26.56% between the VNIR and SWIR portions of the electromagnetic spectrum. In the full-range product generated using the SR^2^ workflow, the shaded canopy VNIR spectrum was consistent with the SWIR; the offset between the VNIR and SWIR portions of the electromagnetic spectrum was <0.3% (see Fig. 8J). These results imply that the large mean absolute offset in the conventional full-range product (7.72% and 7.25% for the forest and urban ROIs, respectively) was due to discrepancies in scale and, by extension, pixel material composition between the CASI-1500 and SASI-640 data. Without applying the SR^2^ workflow, the derived full-range HSI product cannot be effectively utilized for spectroscopy analyses. Similar arguments can be made by studying the building and tree canopy spectra. Although the SR^2^ workflow did not improve the road spectra in Fig. 8I-J, it is crucial to recognize that the extracted pixel was surrounded by other road pixels. As such, the pixel material composition to each spectrum did not change when applying the SR^2^ workflow. This implies that the SR^2^ workflow is critical in heterogeneous areas. Overall, the SR^2^ workflow was shown to be an effective method to ensure that the VNIR and SWIR portions of the spectra from the full-range HSI datasets are representative of similar areas on the ground.

## Concluding remarks and future research directions

In this study, the developed SR^2^ workflow was capable of spatially degrading HSI data based on the spatial characteristics of a coarser resolution sensor. The provided MATLAB script can be used as-is to implement the SR^2^ workflow or adapted as needed. As discussed in the methods section, the developed SR^2^ workflow relies on four assumptions that each impose potential limitations on the approach. These assumptions were necessary to ensure that the workflow is approachable to HSI data end users of all expertise levels. It is, however, important to recognize that the SR^2^ workflow could potentially be improved in future efforts by eliminating some of these assumptions. For instance, for well characterized HSI sensors, the PSF_net_ should account for wavelength dependencies. This could be implemented by modifying the workflow to use a wavelength dependent PSF_opt_. Additionally, the sensor inertial navigation system data could be used to derive a more accurate PSF_mot_. As previously mentioned, a georeferenced raster data input was used as the raster model is effective for data handling and processing purposes [24]. Work from Inamdar et al. [45] show that georeferenced raster end products are negatively affected by resampling errors such as pixel loss, duplication and shifting. Inamdar et al. [45] also present a novel hyperspectral point cloud data format that avoids all resampling errors and preserves spatial data integrity. By using the developed hyperspectral point cloud as a data input instead of conventional georeferenced rasters, the SR^2^ workflow could avoid resampling errors and more accurately simulate the spatial properties of the specified sensor. In this study, we showed the importance of accounting for the sensor PSFs when spatially resampling fine resolution HSI data in data-cross validation, data fusion and flight planning examples. Though the examples from this work only used μCASI-1920, CASI-1500 and SASI-640 HSI data, it is critical to note that the SR^2^ workflow is sensor-independent and can thus be used by other pushbroom hyperspectral imagers. Overall, the SR^2^ workflow was shown to be extremely valuable for downsampling HSI data.

## Direct submission or co-submission

Co-submissions are papers that have been submitted alongside an original research paper accepted for publication by another Elsevier journal.

## CRediT authorship contribution statement

**Deep Inamdar:** Conceptualization, Methodology, Validation, Formal analysis, Investigation, Data curation, Writing – original draft, Writing – review & editing, Visualization. **Margaret Kalacska:** Conceptualization, Methodology, Resources, Data curation, Writing – review & editing. **Patrick Osei Darko:** Conceptualization, Methodology. **J. Pablo Arroyo-Mora:** Methodology, Resources, Writing – review & editing, Writing – review & editing. **George Leblanc:** Resources, Writing – review & editing.

## Declaration of Competing Interest

The authors declare that they have no known competing financial interests or personal relationships that could have appeared to influence the work reported in this paper.

## Data Availability

Data will be made available on request.

## Appendix Supplementary materials

Image, application 1
